# Nanodiamonds in biomedical research: Therapeutic applications and beyond

**DOI:** 10.1093/pnasnexus/pgae198

**Published:** 2024-05-17

**Authors:** Elena Alexander, Kam W Leong

**Affiliations:** Department of Biomedical Engineering, Columbia University, New York, NY 10032, USA; Department of Biomedical Engineering, Columbia University, New York, NY 10032, USA

**Keywords:** nanodiamond, nanotechnology, drug delivery

## Abstract

Nanodiamonds (NDs) comprise a family of carbon-based nanomaterials (i.e. diameter <100 nm) with the same sp^3^ lattice structure that gives natural diamonds their exceptional hardness and electrical insulating properties. Among all carbon nanomaterials—e.g. carbon nanotubes, nanodots, and fullerenes—NDs are of particular interest for biomedical applications because they offer high biocompatibility, stability in vivo, and a dynamic surface chemistry that can be manipulated to perform a seemingly limitless variety of ultra-specific tasks. NDs are already deepening our understanding of basic biological processes, while numerous laboratories continue studying these nanomaterials with an aim of making seismic improvements in the prevention, diagnosis, and treatment of human diseases. This review surveys approximately 2,000 the most recent articles published in the last 5 years and includes references to more than 150 of the most relevant publications on the biomedical applications of NDs. The findings are categorized by contemporary lines of investigation based on potential applications, namely: genetics and gene editing, drug delivery systems, neural interfacing, biomedical sensors, synthetic biology, and organ and tissue regeneration. This review also includes a brief background of NDs and the methods currently developed for their synthesis and preparation. Finally, recommendations for future investigations are offered.

## Introduction

Interest in nanodiamonds (NDs) for use in biomedical applications has grown dramatically in the past decade, with numerous advances reported in the nanomaterial's fabrication, customization, and application (1). Moreover, recent developments have shown proof of concept for the use of NDs in an exciting range of biomedical discoveries and advances in human health (2). For example, NDs may soon facilitate mapping of neuronal activity at axon-specific resolutions or measure neurotransmitter concentrations at specific sites in the brain (3–11).

NDs comprise a family of carbon-based nanomaterials (i.e. diameter <100 nm) with the same sp^3^ carbon-lattice structure that gives natural diamonds their exceptional hardness and electrical insulating properties. The outer surface of a NDs, however, includes unpaired carbon atoms in the sp^2^ configuration, which make the material relatively easy to manipulate to achieve a variety of highly specific objectives. Manipulations of NDs can yield substances with varying degrees of hydrophilicity and colloidal stability, in addition to microscopic tuning of reactivity.

**Fig. 1.**
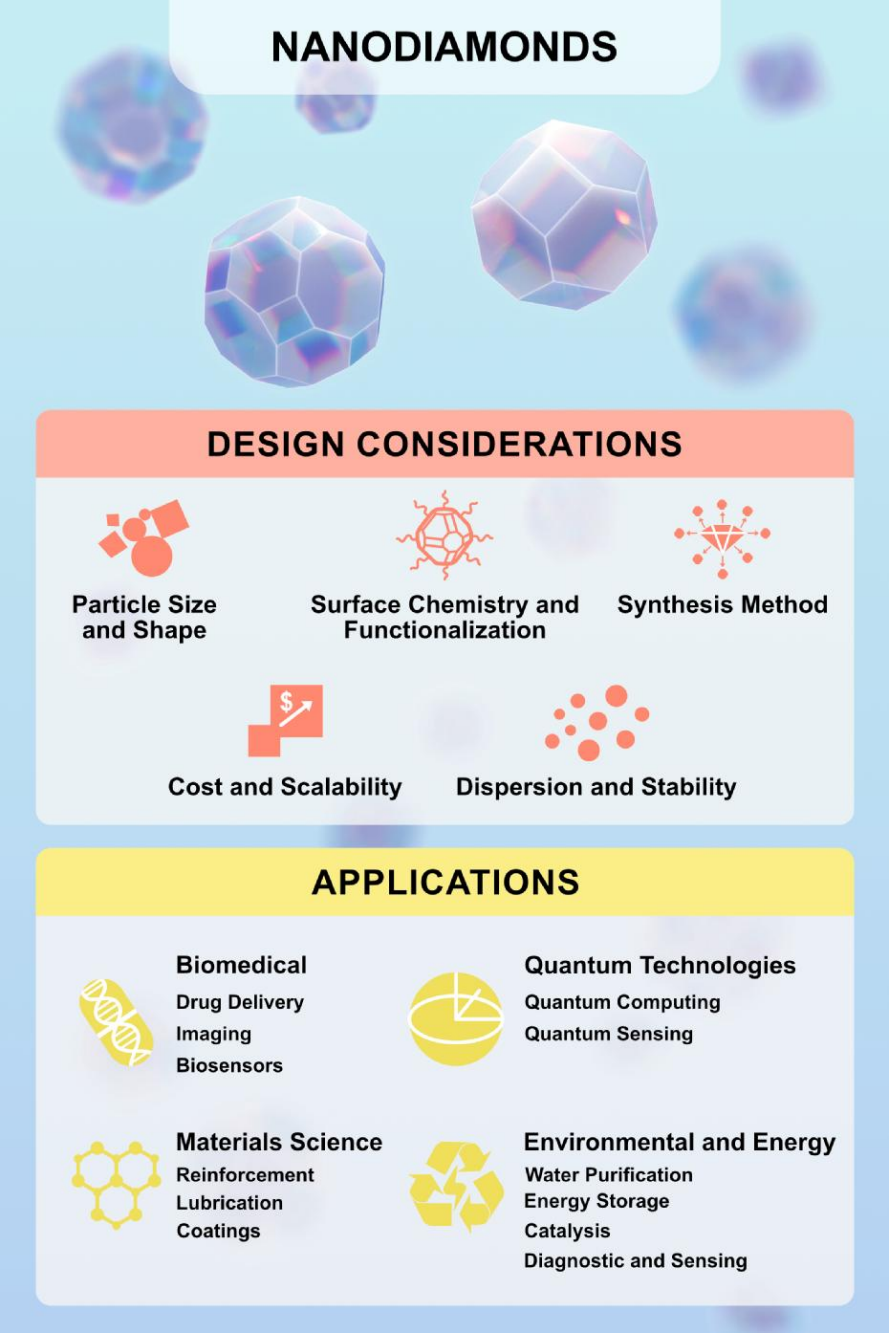
The translational potential of NDs has grown significantly over recent decades, leading to a diverse array of applications with far-reaching impacts in fields ranging from medicine to materials science. Crucial design decisions, originating in research laboratories (e.g. synthesis techniques and surface functionalization), result in ND technologies with distinctive properties, enabling their application across a multitude of translational endeavors. As a ND-based platform progresses beyond the initial stages of research and development, these early design choices lay the groundwork for addressing subsequent manufacturing challenges, ensuring the production of commercial products at scale that adhere to regulatory standards. Original illustration.

Introduction of adulterant chemicals to the process of synthesizing NDs can also generate intentional defects in the sp^3^ lattice structure, which can change refractivity and fluorescence (12). In contrast with other carbon nanomaterials—e.g. nanotubes and fullerenes—which have various arrangements of nonlattice carbon structures, NDs also offer greater biocompatibility and stability in vivo (12–14). Recent studies claim NDs have the highest biocompatibility among all known carbon nanoparticles (15). Figure 1 provides structural diagrams of the various carbon nanomaterials being studied for use in medical research and applications.

This review summarizes the current literature regarding ND research in the biomedical field and provides an overview of emerging discoveries, in addition to recommendations for future research.

### Impact of synthesis methods on ND properties and their biomedical applications

**Fig. 2.**
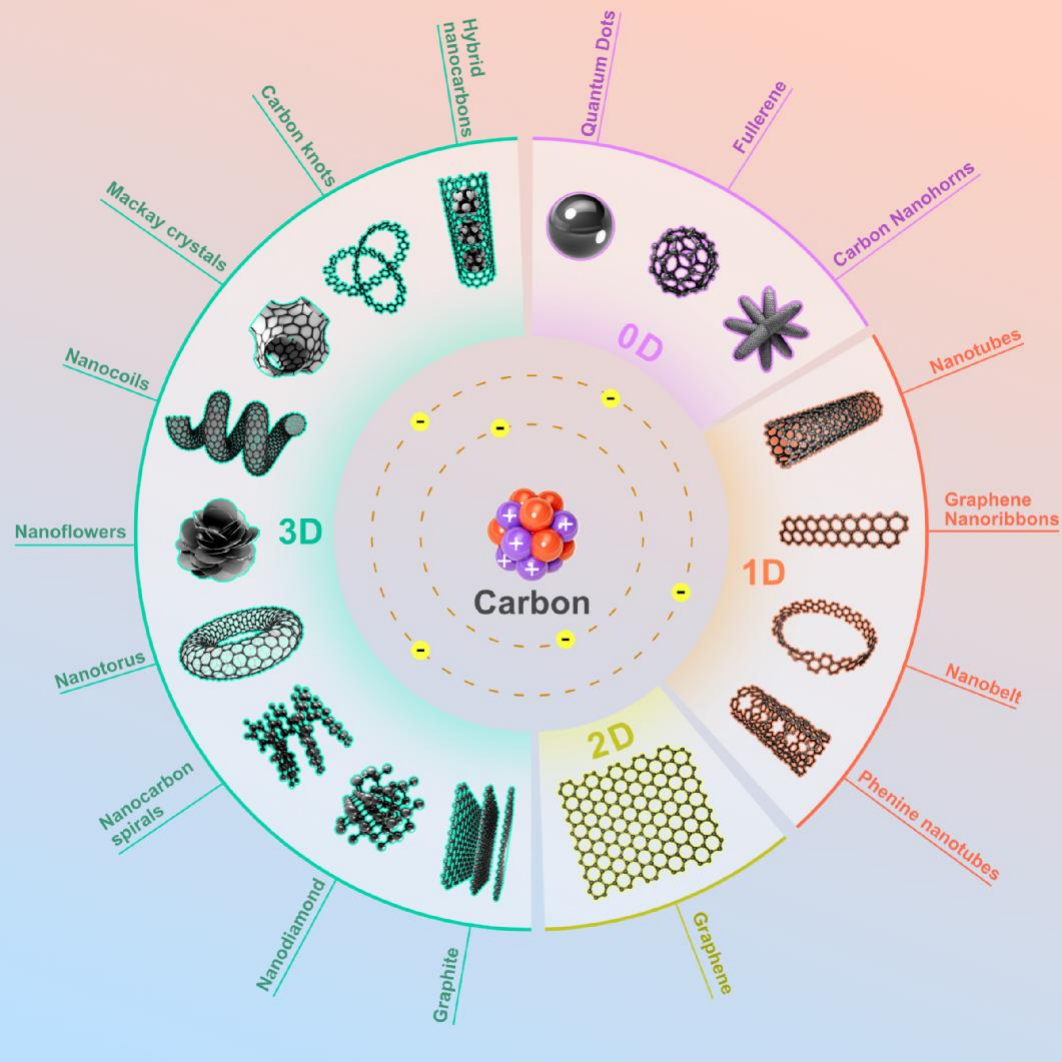
Taxonomy of nano-carbons: a classification of 0D, 1D, 2D, and 3D carbon nanostructures. Each sub-category delineates a distinct dimension, underscoring the versatility and potential applications inherent in these nanostructures within the forefront of materials science and nanotechnology. Original illustration.

The synthesis method significantly impacts the size, shape, purity, and surface chemistry of NDs (Fig. 2), which in turn influences their applicability in various biomedical fields. NDs are typically synthesized through detonation, chemical vapor deposition (CVD), and high-pressure high-temperature (HPHT) methods, each offering distinct advantages and limitations.

Produced by explosive detonation under inert conditions, detonation NDs are typically 4–5 nm in size with a high degree of surface defects. These properties are suitable for applications such as robust free-radical scavengers and drug delivery systems, where high surface area and reactivity are beneficial (16). CVD synthesis allows for the production of ND films and particles with controlled size and fewer surface defects compared to detonation-synthesized NDs. CVD NDs are particularly useful in biomedical imaging and device integration, such as in biosensors and medical implants, due to their uniformity and purity. Their cleaner surfaces reduce nonspecific interactions with biomolecules, enhancing imaging clarity and device reliability (17). HPHT synthesis produces NDs that are typically larger and have fewer surface impurities. These characteristics are beneficial for orthopedic applications where particle stability and mechanical strength are paramount. HPHT NDs are commonly used to enhance the properties of materials used in bone grafts and joint replacements (18, 19).

## Therapeutic applications

**Fig. 3.**
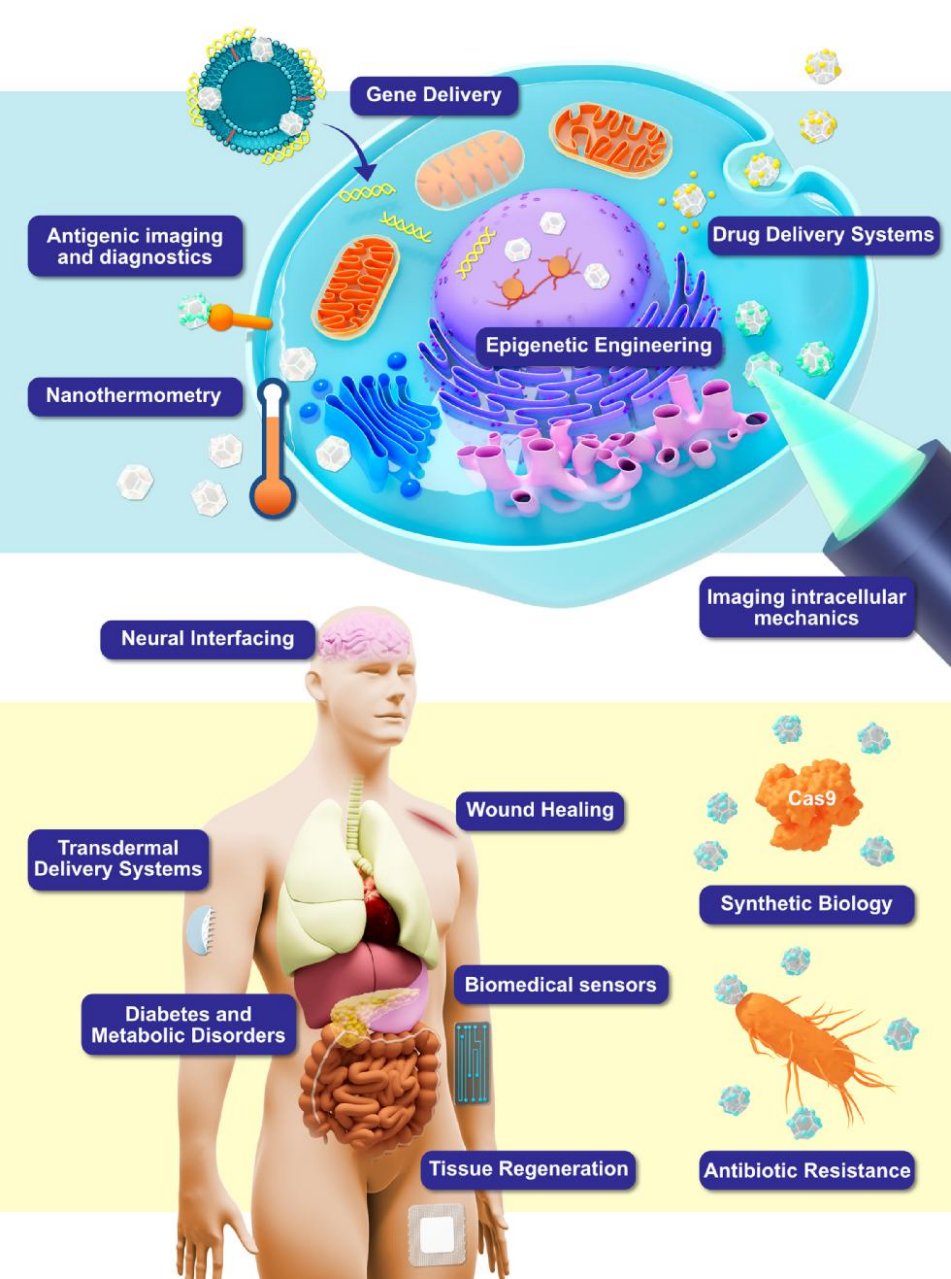
NDs as multifunctional platforms. NDs serve as versatile platforms for various applications, including biomedical and regenerative medicine. Successful ND formulations can accommodate multiple functions, fostering a microenvironment conducive to diverse applications. NDs exhibit intrinsic characteristics, such as biocompatibility and durability, that make them ideal for these roles. They can be engineered to provide mechanical cues for enhanced cellular viability, proliferation, and motility. Additionally, NDs can be designed to evade immune system responses, offering protection for cargo, and they can facilitate signaling to the local environment through the release of bioactive factors. In the context of tissue engineering, NDs also permit the deposition of an extracellular matrix (ECM) by resident cells. These injectable ND-based platforms hold immense potential for addressing a wide spectrum of diseases and injuries. Original illustration.

With very few exceptions, the delivery routes for small-molecule therapies, genetic material (e.g. siRNA), imaging contrast agents, and other medically important compounds have remained largely unchanged for decades. Moreover, the basic routes of therapeutic delivery are generally either manual (e.g. oral/nasal/rectal) or via injection (e.g. subcutaneous/intramuscular/intravenous). However, NDs have been shown capable of entirely new routes for drug administration (e.g. slow-release transdermal patches), in addition to serving as transport systems that can not only release payloads at specific sites but also protect delicate biomaterials, such as RNA and DNA (Fig. 3). The following review summarizes the many ways NDs are being functionalized and investigated for these novel systems.

### Therapeutic gene editing applications

Several studies indicate that NDs possess the potential to serve as an advanced and highly precise delivery platform for CRISPR-Cas9 gene editing (c/c9-NDs). Yang et al. (2020) achieved the successful introduction of a pathogenic mutation associated with X-linked retinoschisis (XLRS)—a point mutation at c.625C > T—into the murine *Rs1* gene by administering NDs conjugated with CRISPR-Cas9 into the mouse vitreous. The team demonstrated both safe and effective delivery of CRISPR-Cas9 components into human induced pluripotent stem cells (iPSCs) in vitro using the ND-conjugated c/c9 system. Importantly, in both experiments, mixing the NDs with bovine serum albumin significantly increased uptake of the c/c9 proteins (20).

The ND particles successfully deliver these components to human iPSCs and mouse retinas, resulting in the introduction of the RS1 c.625C > T mutation. The gene editing in mouse retinas leads to pathological features typical of XLRS. In conclusion, the ND-based CRISPR-Cas9 delivery system demonstrates the potential for creating in vitro and in vivo disease models of XLRS through gene editing (20).

Demonstrating the versatility and potential applications of NDs, Alwani et al. (2022) showed that NDs conjugated with lysine and histidine can form a protective halo around sensitive genetic materials, including plasmid deoxyribonucleic acid and siRNA. Their work confirmed that even these larger conjugations (∼200 nm when fully assembled) can efficiently enter cellular cytoplasm, where the histidine then undergoes acidification and subsequent protonation, disbursing the halo and releasing the protected genetic material for entry into the cell nucleus (21).

### Epigenetic engineering

Epigenetic regulation mechanisms, including DNA methylation and histone modification, control gene expression and contribute significantly to tumorigenesis and propagation. Gu et al. (2019) showed that NDs can deliver epigenetic modulating agents into tumor cells to downregulate genes responsible for tumor growth. In their model of hepatocellular carcinoma, NDs were conjugated with UNC0646, an experimental small-molecule inhibitor of G9a, a histone methyltransferase known to promote tumor progression. This ND formulation showed high efficacy in silencing G9a activity—and therefore, its downstream effects on tumor propagation—when compared with UNC0646 in its liposomal formulation (22).

Many more studies are being conducted on the ND delivery of siRNA to tumor sites using a variety of novel approaches. The purpose of these approaches is to enhance the capacity of fragile siRNA to withstand harsh in vivo conditions and reach cellular cytoplasm in sufficient concentrations to inhibit offending epigenetic activity (23).

### Oncologic drugs

Multiple investigations have utilized NDs as systems to deliver cytotoxic agents directly into tumors. This objective is largely achieved either by tuning the surface structure with pH-sensitive groups that release only when exposed to tumors or making modifications with target molecules that have specific binding affinity for cancer cells (14).

ND technology also opens new avenues for the treatment of tumors that are refractory to standard therapies or located in sensitive regions, such as glioblastomas. Madamsetty et al. (2019) have shown that poly(ethylene glycol)-functionalized NDs offer considerable improvement over free drug in treating pancreatic ductal adenocarcinoma. Furthermore, the team simultaneously loaded irinotecan and curcumin in ultrasmall PEGylated NDs to further enhance the destruction of pancreatic tumor tissue (24).

In addition to their delivery potential, NDs are being investigated for their theranostic capacity—the dual function of identifying cancerous tissue and releasing cytotoxic payloads (13, 14, 25, 26). While this theranostic approach has been applied to multiple disease states, neurological conditions appear to be a particular focus, presumably because of the capacity of NDs to transverse the BBB (27, 28) (see Treatment of CNS diseases section).

**Fig. 4.**
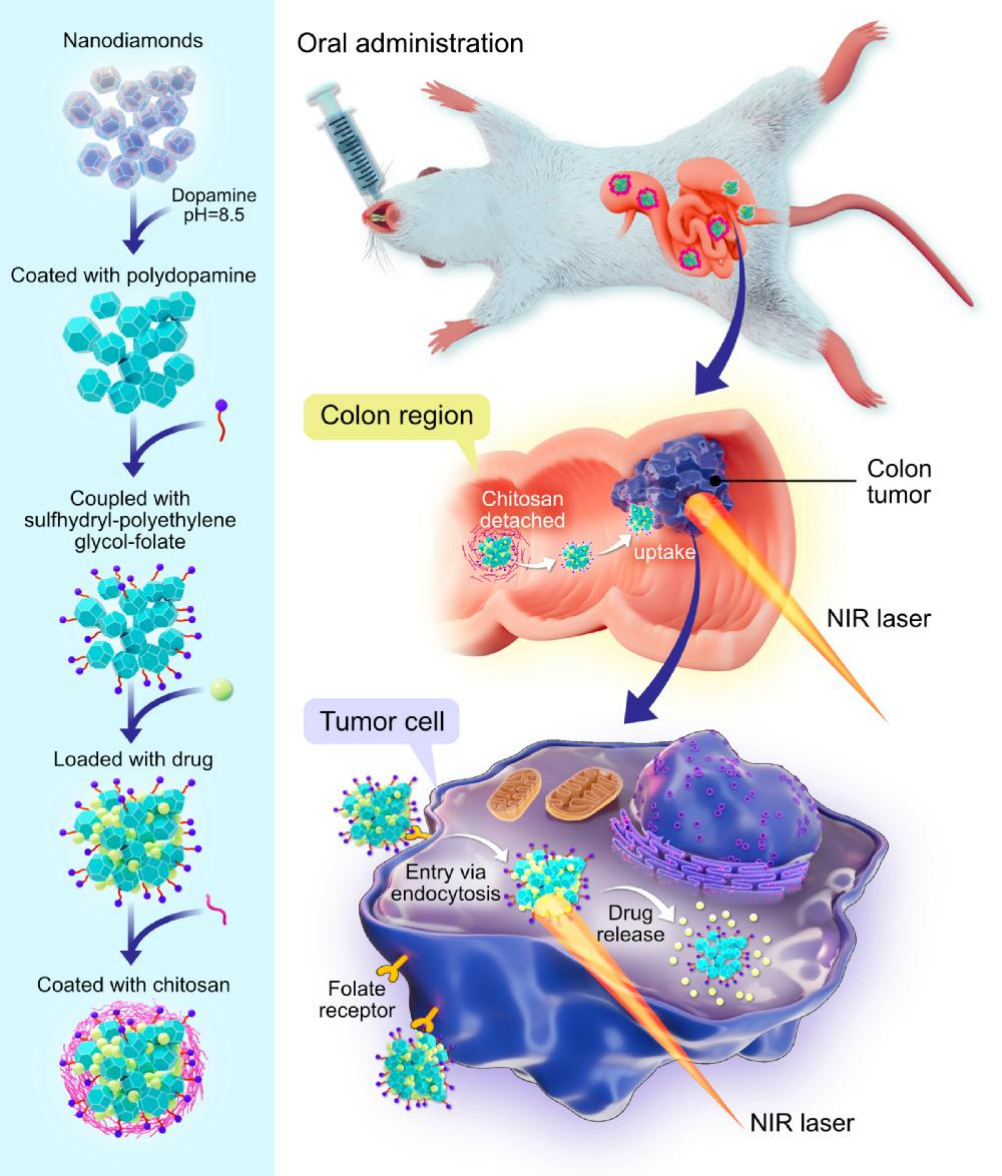
Schematic illustration of preparation of PND-PEG-FA/CUR@CS and the mechanism of combined chemo-photothermal therapy for orthotopic colon cancer by oral administration. Original illustration inspired from the work of Tsukamoto and co-workers (31).

Another dual approach tested in vitro by Li et al. (2022) involves an oral drug delivery system that leverages polydopamine-coated nanodiamonds (PNDs) with colon localization and tumor targeting functions to simultaneously deliver localized chemotherapy and photothermal therapy (Fig. 4). In this work, the PND was used as the photothermal carrier through surface coupling of sulfhydryl-polyethylene glycol-folate, while curcumin was loaded as the model drug, and then coated with chitosan to achieve gastrointestinal tract retention and colon localization (29). The so-called PND-PEG-FA/CUR@CS nanoparticles accumulated in colon tumor cells and displayed high release rates in the target locations when exposed to NIR laser pulses. Similarly, Li et al. (30) conjugated protamine sulfate (PS) functionalized NDs with antiangiogenesis drug combretastatin A4 (CA4) to create CA4-NDs@PS NDs, which demonstrated synergistic antitumor activity in liver cancer by simultaneously releasing the CA4 payload and heating the surrounding microenvironment when exposed to NIR laser pulses.

Lin et al. (2022) developed cetuximab-conjugated fluorescent NDs that could be tracked in real time in human lung tumors xenografted onto nude mice. Their results showed significantly higher uptake of the cytotoxic payload in epidermal growth factor (EGFR)-expressing tumor cells, but not in other cell types (29–31). The ND-Cet complex enhances cellular uptake in EGFR-expressed cells and allows for the real-time detection of EGFR proteins. The therapy aims to inhibit EGFR signaling, which is a common therapeutic approach in lung cancer treatment (29–31).

Tian et al. (32) showed that Fluorescent Nanodiamonds (FNDs) can be dual functionalized to deliver drugs to targeted tumor sites and then illuminate to assess the extent of cell death by optically characterizing the concentration of free radicals at the tumor site.

As an added protection against incidental or unexpected release of cytotoxic agents at sites distant from their tumor targets, or as a method of internally titrating drug release, NDs can be configured into colloidosomes that form a protective sphere around various molecules (33). This measured release has been shown to persist for at least a month or possibly longer (23).

### Immune system engineering

Immuno-oncology represents an exciting field for ND study, and multiple teams have shown the viability of NDs in modulating the immune system and immune response. Suarez-Kelly et al. (34) have shown that FNDs with antibody conjugation can “track and trace” immune responses.

Recent findings from Ho et al. (2021) suggest that negatively charged ND conjugations may have utility as novel antigen carriers for vaccine development. Their team conjugated Detonation Nanodiamonds and HPHT-NDs with a plant-based hemagglutinin protein (H5.c2) of A/H5N1 influenza virus and showed that surface change of the resulting particles has wide implications for both functionalization and in vivo behavior (35).

### NDs for drug delivery to stem cells

**Fig. 5.**
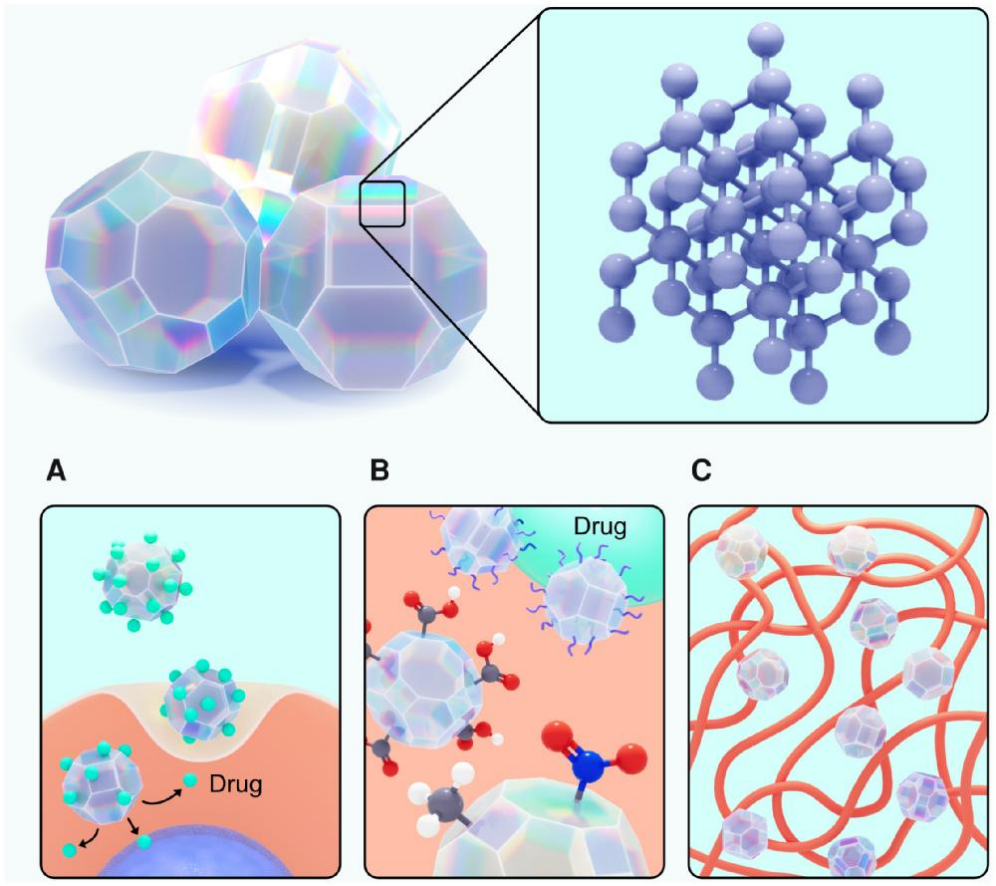
Key structural attributes of NDs. NDs, characterized by their nonporous crystalline lattice structure, exhibit unique structural features influenced by synthesis methods and surface modifications. These structural attributes, such as surface defects and dopants, hold significant implications for various applications, including drug delivery and quantum technologies. ND-based drug delivery systems. A) NDs can function as hybrid drug delivery systems, combining their inherent properties with drug encapsulation and delivery capabilities. B) Surface modifications of NDs enable the controlled release of drugs, particularly in cases where the drugs are larger than the NDs themselves, by adjusting the surface chemistry or functional groups. C) NDs can also be integrated into other materials, such as polymers, to create composite drug delivery systems, offering versatile options for tailored drug release and targeted therapies. Original illustration.

Much work has sought to leverage the drug delivery (Fig. 5) benefits of NDs specifically to direct the fate of stem cells in the treatment of numerous diseases (36). For instance, techniques are being developed to repair tissue loss in lung diseases using ND-directed stem cell therapy (37). Other approaches deploy NDs conjugated with cytotoxic chemotherapy to target and destroy cancer stem cells, a technique that was shown to be highly efficacious in vivo, even exhibiting high cytotoxicity among cancers that have acquired resistance to various chemotherapies (38).

Liu et al. (2021) recently showed that NDs can serve as efficient transporters for microRNA. Specifically, the team functionalized NDs to transfect iPSCs with MiR-181a and directs the cells to differentiate into cardiomyocyte lineage, an approach with significant implications in the recovery of heart cells lost to infarction or other damage (39).

In 2019, Taylor et al. (40) demonstrated that oxygen-functionalized ND monolayers yield a high degree of neuronal differentiation and network formation in vitro, suggesting it as a promising platform for stimulating growth of neurological tissue.

## Diagnostic applications

Despite regular advances in the understanding of microbiological interactions that regulate and support life, experimentation and discovery remain hampered by certain technological limitations. These include difficulty observing and tracking the movement of proteins among and within cells, which is complicated by the environmental dynamics that govern cellular and organelle interaction with various molecules and with each other. Moreover, the interplay of these interactions is at the center of health and disease. The use of NDs—and FNDs in particular—as targeted imaging agents holds the tantalizing potential to elucidate many of these mechanistic interactions for the first time. The following review summarizes the current state of the science in this regard and highlights key areas of development.

### Neural interfacing

**Fig. 6.**
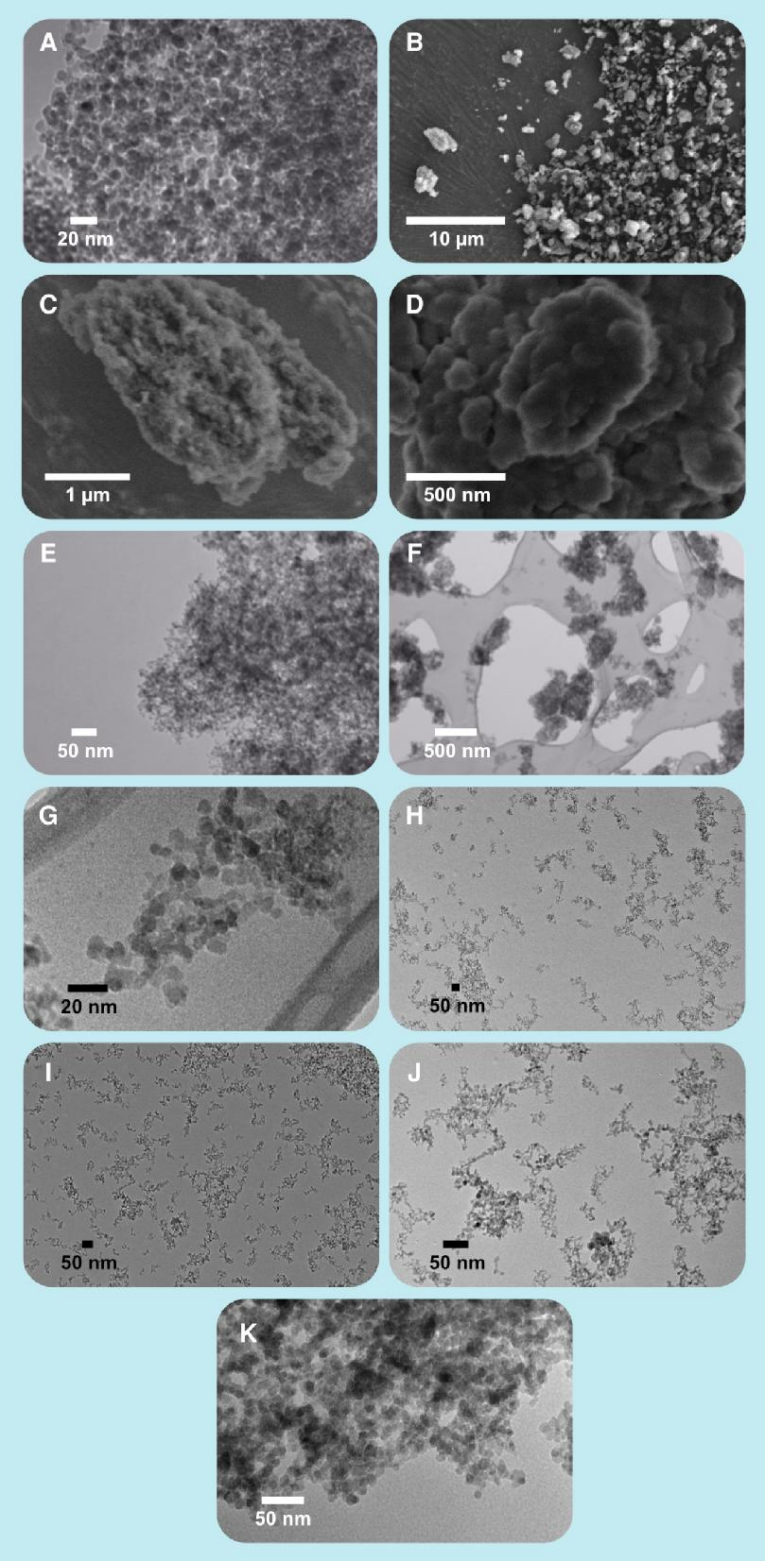
Images of NDs at various resolutions as they appear when captured with scanning-electron and transmission-electron microscopes. Laser synthesized NDs. Images (A), (E), (F), and (G) Transmission Electron Microscopy. Images (B), (C), and (D) SEM. Original illustration and images.

The development of implantable nanoscale neuromodulation technologies holds great promise for both resolving central nervous system (CNS) activity in vivo and treating a host of neurological and psychiatric conditions. There are a multitude of diseases to be addressed with just a handful of examples ranging from macular degeneration to depression to Alzheimer's disease and stroke. NDs have been proposed and investigated as leading candidates for these applications, largely because of their ability to cross the BBB and their capacity for multifunctionalization—i.e. perform imaging, diagnostic, and drug delivery functions. Additionally, nanoscale biomaterials (Fig. 6) have the potential to overcome a host of current technological hurdles that limit researchers’ ability to develop and understanding of brain function at a high resolution on the cellular and neurotransmitter level. The following review summarizes the current state of research into leveraging NDs to deepen our understanding of neurological function and treat disease.

#### Neural imaging and neuronal dynamics

Because of their high sensitivity to electrical and magnetic stimuli, FNDs have been proposed as bright and photostable quantum probes to evaluate neuronal activity in near-real time (41). Demonstrating proof of concept, Moscariello (42) proved that FNDs stabilized by a protein-derived biopolymer coating (cationic, PEGylated denatured human serum albumin; dcHSA-PEG) not only cross the BBB in vivo in mouse models but also are readily taken-up by individual neurons.

In addition to monitoring neuronal activity in vivo, other researchers have suggested developing ND probes that would provide a window into the concentrations and interactions of neurotransmitters (43). Work in this regard is actively developing. Shao et al. (2022) developed ND electrodes that reliably and consistently detected dopamine in mouse brain tissue in vitro. Their report showed that the ND dopamine probes showed high sensitivity for dopamine and high resistance to fouling and noise from serotonin and other electrochemical stimuli (9). Similarly, Puthongkham and Venton (8) showed that treating carbon-fiber nanoelectrodes with ND surface coating increased the reliability of a dopamine signal and decreased fouling of the electrode from environmental contaminants.

Because of the dramatic differences in regional brain function, targeting specific neuronal types is imperative (7). Petrini et al. (2022) have shown that FNDs can be functionalized not only to cross the BBB but also to target hippocampal neurons. Their work has implications in understanding neurological disease processes and featured a neuronal nano thermometer with a specificity of at least 1K. Results of their in vitro experiments showed ND nanothermometers can predict changes in neuronal activity and metabolism (7).

Drug delivery into specific neurological cell types also holds promise for more targeted therapies. To this end, Parker et al. (6) coated fluorescent NDs with various lectin proteins that have targeted binding affinities for different glycan receptors on CNS cells. Neurons, microglia, and astrocytes successfully endocytosed these FNDs, which remained in the cells for ≥48 h with minimal stress to the host cells.

Other, more traditional uses for FNDs include novel contrast agents for use in magnetic resonance imaging (MRI). In a series of investigations, Panich et al. (4, 5) demonstrated in rats that saline suspension of polyvinylpyrrolidone-coated gadolinium (Gd[III])-grafted NDs provides significantly higher signal intensities than the common tracer gadoterate meglumine. The team has found similarly promising results using manganese-grafted ND as contrast agents (3, 5).

#### Diagnosis of CNS diseases

A proven deployment of functionalized FNDs is in the detection of biomarkers for Alzheimer's disease (28). Specifically, NDs can be functionalized with a cell-penetrating peptide (R7) and a beta-sheet breaker peptide that could locate and bind to amyloid-beta plaques in vivo in Alzheimer's disease mouse brains (28, 44).

Gheorghe et al. (45) validated a biomarker for brain tumors using protopotphyrin IX, immobilized in ND paste, as a sensor for interleukins 1β, 6, and 12 from several biological samples, including whole blood, urine, and tumor tissue. Their screening system uses a needle stochastic sensor to detect brain cancer from whole blood samples with recoveries >96% (± < 1%).

Others have proposed leveraging NDs to enhance neuronal growth and regeneration. For example, Chen et al. (2020) leveraged ND surface topography to grow neuronal tissue ex vivo and subsequently discovered that the ND environment depleted the previously uncharacterized microRNA (miR6236) while enhancing neuronal growth. Their studies confirmed that miR6236 is the predominant molecule responsible for converting surface topography into biological responses and that its depletion enhanced neuroregeneration on an inhibitory substrate (46).

Applications of nanothermometry (see Nanothermometry section) are already impacting understanding of the effects of temperature on neuronal activity. Petrini et al. (7) leveraged NDs to quantify the correlation between intracellular temperature variations and the modulation of neuronal activity in *Caenorhabditis elegans* worms.

#### Treatment of CNS diseases

**Fig. 7.**
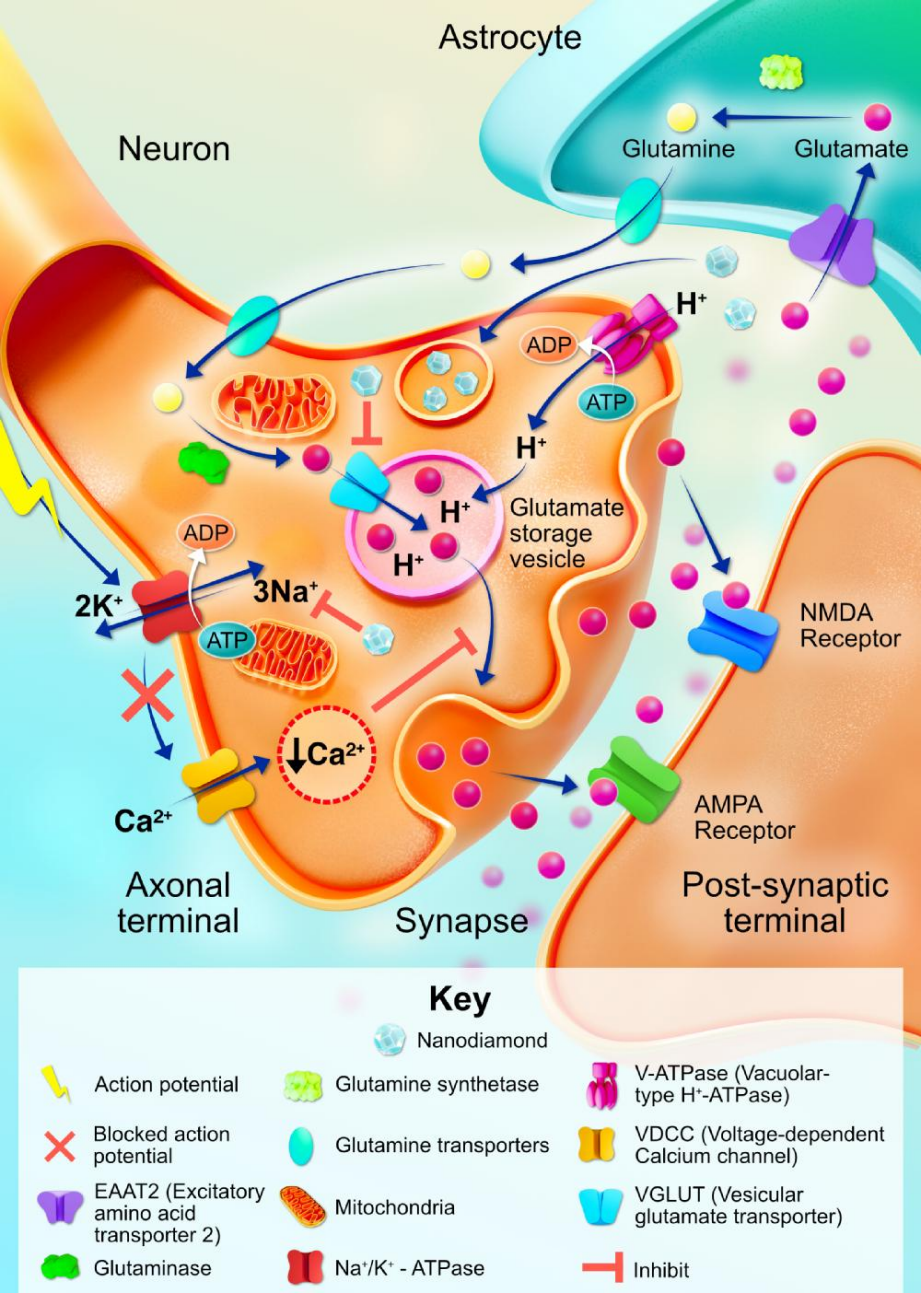
The ability of NDs (NDs) to alter and/or modulate neurotransmission signaling in neurons. Once taken up by neurons, NDs are proposed to influence and hinder the vesicular glutamate transporter located within the axonal terminal and the sodium–potassium (NA+–K+)-ATPase located on the membrane. This interference appears to reduce the amount of glutamate stored in vesicles as well as resulting in the decreased release of glutamate at the synapse. Therefore, NDs could curb the glutamate excitotoxicity often observed in many neurological disorders. Original illustration inspired from the work of Saraf and co-workers (28, 49, 51).

NDs have a well-established capacity to cross the BBB (Fig. 7) (27, 28, 47–50). Moreover, NDs have been shown in vivo capable of transporting larger therapeutic agents across the BBB when the agents suspended in serum are less likely to reach their target in the CNS (28, 47, 48). This important characteristic makes them especially attractive candidates for the treatment of CNS diseases (28, 41).

Functionalization of NDs for the treatment of CNS diseases has been achieved through a variety of different approaches. Using carbodiimide chemistry, Ghanimi Fard et al. (2022) functionalized FNDs with high specificity and selectivity for specific various glycans on the surface of different types of brain cells. The FNDs were taken up in neuronal, microglial, and astrocyte cells in mouse brains (52). Taking a different approach, Al Qtaish et al. (51) functionalized NDs with niosomes to both transport genetic material across the BBB and protect the vulnerable material from degradation while exposed in vivo.

One line of investigation into potential therapies for Alzheimer's disease and stroke focused on the conjugation of NDs with amlodipine, a calcium channel blocker that cannot pass through the BBB but is theorized to have neuroprotective effects. Specifically, amlodipine may that reverse the calcium-induced excitotoxicity and mitochondrial dysfunction, both of which underlie several neurologic disorders (53).

Using NDs conjugated with polydopamine and indocyanide green, Maziukiewicz et al. (54) showed in vitro that targeted photo excitation of functionalized NDs can be locally cytotoxic to glioblastoma cells while sparing most surrounding tissue.

#### Neural prosthetics and implants

Restoration of sight represents one avenue of investigation for NDs (55). Stamp et al. (56) created a retinal implant with ND pillars that stimulated retinal ganglion cells in rats at 20 times the strength and accuracy as existing implant technologies.

In a potential application of the neurotransmitter detection system discussed in Neural imaging and neuronal dynamics section, Cao et al. (57) reviewed the potential for carbon nanomaterials—including NDs—to be functionalized into nanoprobes that would detect neurotransmitters over longer periods of time. The review reports that many investigations have demonstrated nanoprobes can be viable in vivo over the short term, although few have studied their long-term implantation and use.

Guarina et al. (10) have demonstrated that FNDs could be deployed to assess neuronal activity in vitro in hippocampal cells. Hejazi et al. (58) proposed a hybrid ND-carbon fiber nanoprobe that could monitor and modulate neuronal action potentials to optimize dopamine levels. Olăreț et al. (59) incorporated NDs into tubular scaffolds that showed superior nerve regeneration along specified pathways, with cases of trauma or surgical intervention being of particular interest.

#### Neuromodulation

Multiple implementation paradigms have shown the capacity for functionalized NDs to affect neuronal activity in vivo (41, 60, 61). For example, Jiang et al. (62) showed that NDs can be configured to affect neuronal activity by manipulating cytoskeletal transport and polymerization in neuronal cytoplasm. Other research has shown that NDs can modulate membrane potential across synapses, thereby affecting neurotransmitter uptake (63, 64). A different paradigm for the use of NDs to alter neuronal activity leverages their capacity to assemble into superstructures with a high density of implantable nanoelectrodes. Stamp et al. (56) are currently investigating this paradigm for implantation into the retina to address visual degeneration.

### Biomedical sensors

Owing to their high photostability and biocompatibility, NDs have been the focus of myriad development programs in biosensing. At the macrolevel, examples of this work include the development of wearable biosensors that can monitor a litany of health indicators. At a molecular level, potential applications include high-resolution imaging and thermometry to deepen understanding of intracellular mechanics and the presence of various disease biomarkers. The following review summarizes the current areas of investigation for deployment of NDs as highly sensitive agents for exposing biological processes with unprecedented detail and fidelity.

#### Oncologic imaging

As previously mentioned, ND technology is undergoing intense study in oncology. This includes deployment of functionalized NDs for the detection and analysis of cancer and tumorigenesis. Among the advantages offered by FNDs is their ability to identify specific proteins known to play a role in tumor development and metastasis (31). For example, Lin et al. (2022) conjugated FNDs with cetuximab to track in vivo the location and distribution of EGPR, a protein that plays a crucial role in tumorigenesis and is a key target for the treatment of lung cancer (31).

Similarly, Jariyal et al. (65) reported the application of FNDs in detecting the presence and density of cancer stem cells, a cancer cell type that is highly metastatic, resistant to chemotherapy, and difficult to detect because it is found primarily in the core of solid tumors.

Zhou et al. (2020) developed a “smart” fluorescent ND with reversible and highly sensitive zwitterionic charge. In the presence of tumor pH (slightly acidic), the FND exhibits a positive charge, whereas at pH above 7.4, the FND exhibits a negative charge. This adaptable surface charge offers a potential imaging system with high affinity and sensitivity to tumor cells (66).

#### Resolving intercellular dynamics

Cells live and function in a highly dynamic environment that varies in ways that significantly influence cellular function (67). NDs are being investigated as tools to deepen understanding of this microenvironment, including factors such as pH, temperature, and traction (27, 68). While nitrogen-vacancy FNDs are well known for their photostability and imaging properties, silicon-vacancy (SiV) FNDs offer complimentary properties of distinct dispersion patterns throughout cells and different spectral bands (69). With this in mind, Bray et al. (70) proposed a novel multicolor labeling system that leverages the unique properties of NV and SiV NDs. However, deployment of SiV NDs with in vivo applications remains hampered by a much lower density of vacancies than NV-NDs by a factor of 100 or greater (71).

Other color centers have also emerged as candidates for imaging, including germanium, tin, and lead, all from group IVA of the periodic table below carbon and silicon (72).

There is a particularly large gap in our understanding of the mechanics of the lymphatic system, which is largely too selective to take-up MRI contrast agents and therefore difficult to visualize. Yano et al. (73) functionalized FNDs with a gadolinium-based contrast that allowed for MR imaging of lymphatic systems in vivo and in real time.

#### Imaging intracellular mechanics

Comprehending the complex dynamics of organelles, proteins, enzymes, and other functional materials within individual cells remains a difficult undertaking (67). As evidence of the capacity of NDs to elucidate processes involved in intracellular dynamics, Liu et al. (69) created a SiV-FND (as opposed to nitrogen-vacancy) that allowed for dual-color imaging and intracellular tracking of functional components in a live cell.

An application of FNDs with broad appeal for investigating these intercellular interactions is relaxometry, a process that utilizes a strong laser pulse to polarize the spin of a NV-ND. When the laser is switched off, the FND exhibits stronger fluorescence than what is detected at equilibrium, allowing for time-based monitoring of its movement and decay (74).

Importantly for understanding neurodegenerative diseases, FNDs have been used in mouse brains to monitor intracellular movement and transport of alpha-synuclein and beta-amyloid, which are implicated in Parkinson's and Alzheimer's diseases, respectively (75). Moreover, Pelicci et al. (76) have shown that NDs can be deployed to quantitatively map specific regions of interaction within a cell, measuring at the nanoscale level the relative spatial distribution of different molecular species. Similarly, Schmidheini et al. (77) developed a super-resolution microscopy system of FNDs functionalized with gold nanoparticles (GNPs) that generates stochastic blinking capable of monitoring movements and activities of intracellular organelles.

#### Free-radical studies in cellular dynamics

A specific area of focus in understanding intra- and intercellular dynamics is the study of proteomics. However, the production and role of free radicals have proven elusive because current methodologies rely on chemical reaction detection, which can be masked by low signal-to-noise ratios (78). In response to this, FNDs have been functionalized for relaxometry (see Imaging intracellular mechanics section) and have been able to detect and track nitric oxide and superoxide with a precision of approximately 10–20 nm (78). FNDs offer the potential to greatly expand knowledge about the interactions occurring within cells and organelles because FNDs are readily taken up by and can be tuned to have for affinity for specific proteins or genetic materials (67, 68).

Other work has leveraged NDs to monitor effects of free-radical modulation on individual macrophages, as radicals are thought to play an important role in immune function (79).

Along this same line of investigation, Wu et al. (80) developed a ND-based relaxometry system that showed in vivo an uptick in free-radical production in mice early in a viral infection and during replication. Similarly, other groups are functionalizing FNDs to detect the presence and concentration of important metal cations, such as sodium (Na^+^) and potassium (K^+^), both of which are critical to cellular function and thought to be dysregulated in some disease states (81). Additional work has focused on high-resolution detection of other important biochemicals, such as L-ascorbic acid (82) and glucose (83). Similar translational work has assessed ND-based relaxometry to assess the free-radical load in synovial fluid of patients with arthritis. This work from Elías-Llumbet et al. (84) successfully distinguished between patients with osteoarthritis vs. rheumatoid arthritis and proposed a mechanistic explanation for the higher efficacy of the nonsteroidal anti-inflammatory drug (NSAID) piroxicam among patients with osteoarthritis than those with rheumatoid arthritis. These lines of investigation are also relevant in toxicology, where studies have utilized FNDs to reveal free-radical production and concentrations in individual organelles of liver cells after exposure to toxic levels of acetaminophen (85).

Importantly, it has been shown that FNDs are unlikely to produce prolonged oxidative stress in cells after internalization and therefore can serve as passive monitors for the presence and movement of free radicals within individual cells (86).

#### Antigenic imaging and diagnostics

Rapid detection of viral exposure and infection represents a promising application for FNDs (87, 88).

Wei-Wen Hsiao et al. (88) showed that a competitive diagnostic system using FND for visualization could detect the presence of specific SARS-CoV-2 strains with more than 20 times the sensitivity of current technologies. Similarly, gold-conjugated ND sensors have been shown to detect SARS-CoV-2 spike protein using electrochemical impedance quickly and with high accuracy (89).

Miller et al. (2020) recently developed a model FND-based detection system that they theorize could be deployed at the point of care for rapid identification of exposures and infections. Their system could detect the presence of HIV RNA material with a 50-fold greater sensitivity than they World Health Organization's threshold of 1,000 copies/mL (90).

Heish et al. (2019) demonstrated a system for determining the position and density of antigens on human cells using modulated FND detection. The results aligned with an assay of R-phycoerythrin-conjugated antibodies by flow cytometry, supporting the reliability of this approach (87). Similarly, FNDs have been shown viable for rapid detection of allergens in foods using a ND-based voltammetric immunosensing platform (91).

Among the more novel applications of ND technology is genetic sensing based on NDs with a nitrogen vacancy (NV) center. Li et al. (92) recently demonstrated the capacity of NDs to detect the presence of SARS-CoV-2 RNA via an optically detectable magnetic noise signal. The team demonstrated consistent detection of the viral RNA with a very high sensitivity—down to a few hundred RNA copies—with a false negative rate <1%. Additionally, they suggested that ND-based RNA sensors could be tuned with different defects and substrates to diagnose other RNA viruses.

Using NDs again but with a different approach, Zhu et al. (93) showed that spermine-modified NDs can selectively enrich oligonucleotides to assist in detecting human papilloma virus with mass spectrometry. This research was performed using real-world clinical samples, and the authors opined that a similar process could also enhance mass spectrometry analysis of DNA methylation, single-nucleotide polymorphisms, and other vital genotyping.

#### Nanothermometry

NV-NDs represent dynamic sensors capable of detecting several parameters, including magnetic and electrical fields, temperature, pressure, nuclear spins, pH, and deformation (94). In particular, their spin resonance frequencies vary with temperature, making them a promising thermometer. The high thermal conductivity, high hardness, chemical stability, and biocompatibility of NV-NDs enable reliable and fast temperature sensing in complex environments, such as live systems (95–97). Systems have been developed that can monitor temperature changes in vitro as low as approximately one-fifth of 1°C (94) and over the course of at least several days (98). Other researchers have suggested using ND thermometers to monitor wound healing, with minute elevations in temperature correlating with infection (94, 99).

Multiple lines of study are investigating methods to combine temperature sensing and temperature modulation in a single ND-based structure. For example, Sotoma and Harada (100) developed a novel ND system of FNDs functionalized with GNPs, polydopamine (PDA), and hyperbranched polyglycerol to create a system capable of both monitoring local temperature and performing localized heating when photostimulated. Wu et al. (101) developed a local heat monitor and generator using NDs coated with a nanogel shell and indocyanine green. Dou et al. (102) developed a highly sensitive nanothermometer to monitor internal battery performance with translation potential into cellular monitoring.

SiV-FNDs also offer high-speed resolution and temperature sensitivity. Choi et al. (103) performed parametric analysis on the photoluminescense of SiV-FNDs to propose a nanothermometer capable of detecting a 0.4°C change within 0.001 s.

Other similar approaches are investigating the potential of NDs to probe in vitro pH of various systems. Padrez et al. (104) developed an FND system in which NDs photoluminescence increases according to the pH of it surrounding environment. Fujisaku et al. (105) developed a similar system in which FNDs conjugated with carboxyl groups could measure pH ranges in various biological media (21).

Another possible application for NDs in biomedical research is development of gas-sensing media that can be modified for sensitivity to target compounds. Li et al. (106) developed a multifunctional CVD-ND system with sensitivity for any of hydrogen gas (H_2_), ammonia (NH_3_), or ultraviolet light. Schmidheini et al.'s (77) work (discussed also in Imaging intracellular mechanics section) included coupling of stochastic-blinking FND with gold-nanoparticles that detected and visually tagged fluctuations in temperature over time.

**Fig. 8.**
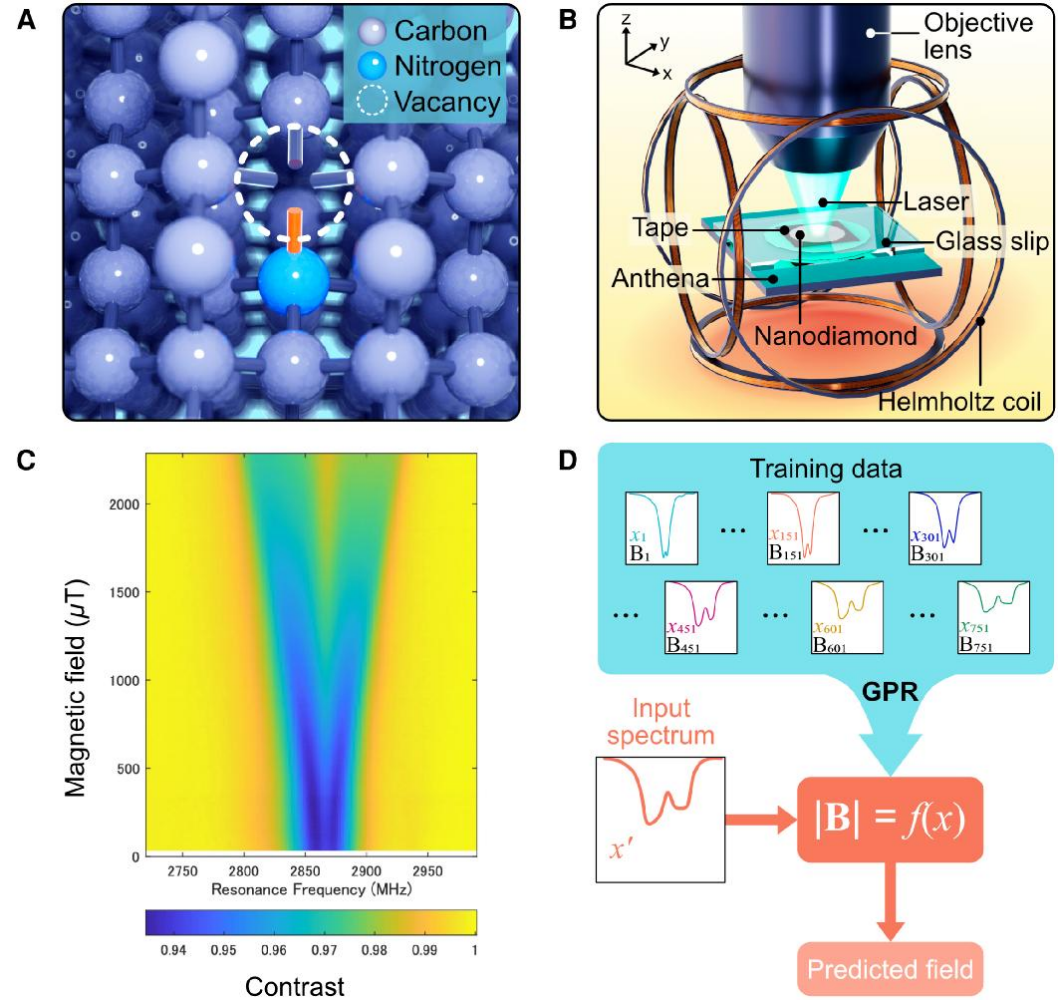
Implementation of ND quantum sensors enhanced by machine learning. A) Schematic of a nitrogen-vacancy (NV) center in diamond. B) Experimental setup. The optical axis is the z-axis, and the NDE is spread on the surface in the xy-plane. C) Experimentally obtained ODMR spectra of NDE as functions of the microwave frequency and the magnetic field. The true magnetic field is measured using a tesla meter. D) Schematic of our machine learning method. The ODMR spectrum and the true magnetic field of (C) are used as the input vector $x_{i}$ and output scalar $y_{i}$ for training, respectively. Using GPR, a function is obtained from the training data to predict the magnetic field strength $|B|=f(x{)}^{\prime }\;$ from an unknown spectrum $x^{\prime }$. Original illustration inspired from the work of Tsukamoto and co-workers (108).

Important parallel work in nano-biosensing seeks to leverage machine learning techniques (Fig. 8) to better collect and interpret data from FNDs (107).

#### Wearable devices

NDs have been shown to enhance nanomaterial configurations designed for use as wearable devices. In one instance, researchers incorporated NDs into graphene fiber to enhance the flexibility and stretchability of the graphene sheets. The “molecular-level lubricating” effect of NDs allowed graphene fiber to retain the same electroconductive properties as unconjugated graphene without the deleterious effects of dielectric additives, such as polymers, that are currently deployed in most graphene-fiber configurations (109).

**Fig. 9.**
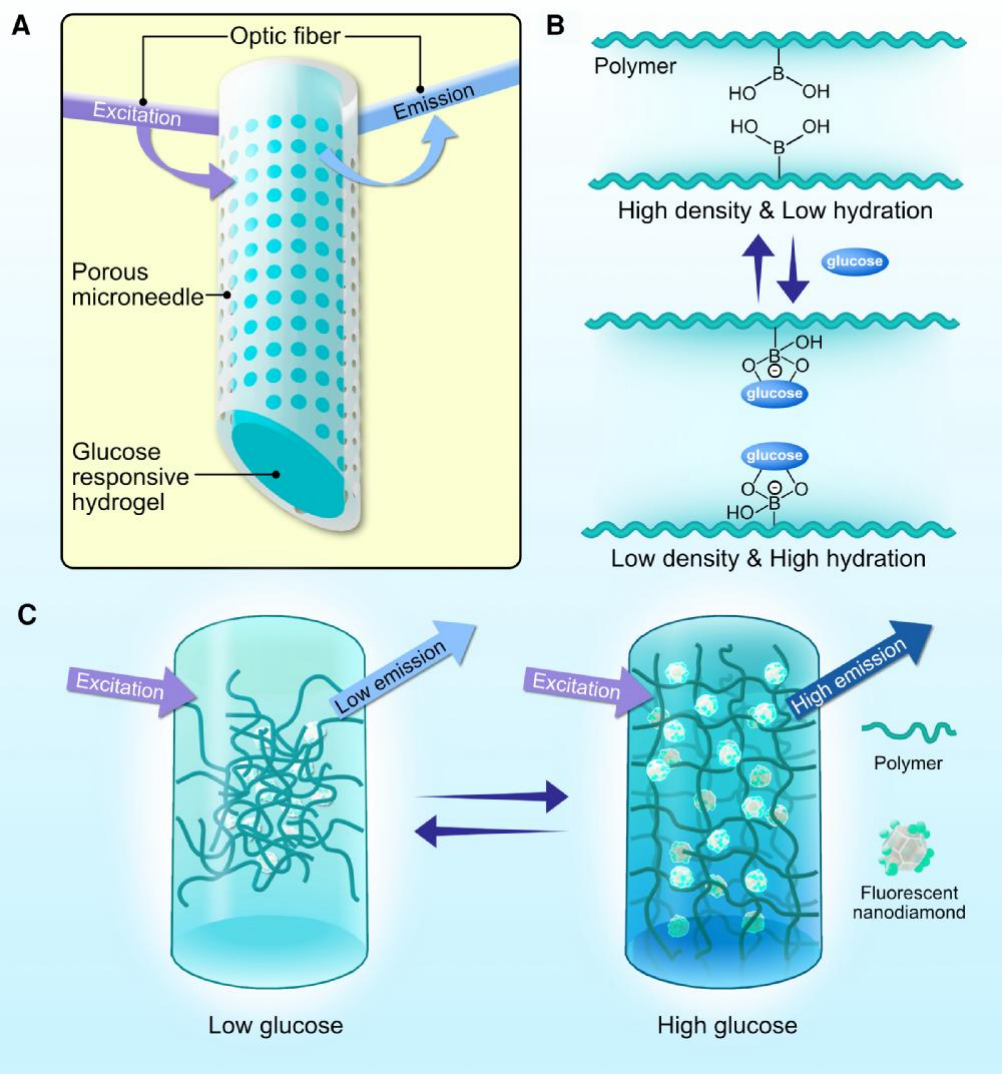
Schematics of the microneedle with fluorescent ND-based hydrogel. A) Schematic illustration of the microneedle device comprising transparent porous wall and covalently bound fluorescent ND-based boronic hydrogel for glucose sensing. B) The reversible complexation of boronic acid group and cis diols of glucose molecules enables the reversible changes of the polymer network at different glucose concentrations. C) Schematic illustration of the glucose-responsive hydrogel that can change fluorescence output according to glucose concentration. Original illustration inspired from the work of Zhang and co-workers (110).

In another widely applicable device concept, multiple lines of investigation are evaluating ND configurations for continuous glucose monitoring (CGM). Zhang et al. (2023) developed a fluorescence-based CGM that leveraged FNDs suspended in a hydrogel and incorporated into a microneedle. Their prototype system successfully measured glucose levels (Fig. 9) in free-moving pigs for several days with no signs of irritation at the microneedle site (108). Furthermore, the authors theorize that the NDs in the hydrogel could be configured to perform monitoring for many different diseases and conditions ranging from cancer progression to epilepsy (108).

## Biomedical engineering applications

Medical researchers have a keen interest exploring in novel uses for nanomaterials, including NDs, to enhance the provider's ability to diagnose and treat diseases. For the purposes of this review, we loosely describe biomedical engineering in the context of manipulating tissue and genetic materials, acknowledging its significant impact on medical care.

### Synthetic biology

Broadly speaking, synthetic biology encompasses a field of science that involves redesigning organisms for modified purposes by engineering them to have new abilities (111). While applications in this well-established field touch upon everything from bioremediation to agriculture, NDs have been proposed to facilitate and enhance the development of fully or partially synthetic materials for use in the detection and treatment of human diseases. The following review summarizes these potential applications.

#### Synthetic proteins and bioprinting

The use of nanoparticles to block specific protein–protein interactions is emerging as an alternative to small molecule-based therapies. However, the nanoparticles designed as “artificial proteins” generally require surface modifications to ensure selectivity, which NDs have been shown to facilitate (110). Specifically, Balek et al. (110) used ND coatings to enable specific and efficient targeting of fibroblast growth factors without interfering with other growth factor systems and serum proteins.

Advanced bioprinting of injectable and bioprintable hydrogels has also advanced rapidly, and the inclusion of NDs into these media can improve not only their biocompatibility but also their stability and compressive strength. Bhattacharyya et al. (112) demonstrated that ND particles improved compressive strength nearly 10 times over unmodified hydrogel while simultaneously improving cellular interaction with the gel and its stability in water.

### Tissue regeneration

Regenerative medicine has evolved rapidly over the last 10–15 years, owing in large part to the discovery of iPSCs, which are a modified version of adult stem cells that have been reprogrammed to a state very similar to embryonic cells. Although there have been important and undeniable advances in regenerative medicine, such as chimeric antigen receptor T-cell therapy for hematological malignancies, widespread implementation of regenerative technologies has been hampered by a litany of limitations. For example, the restoration of neurons lost to stroke or cardiac tissue damaged from a heart attack remain unrealized promises of this dynamic field. Ultimately, the goal of any regenerative treatment is to replace or repair lost or damaged tissue, thereby regaining tissue/organ function. The following review summarizes and highlights the important role NDs are playing in newly envisioned strategies for both in vivo and in vitro tissue regeneration.

#### Neural regeneration

Several lines of investigation are evaluating the capacity of various ND configurations to direct the fate of human neural stem cells (40). In 2019, Taylor et al. (40) demonstrated that oxygen-functionalized ND monolayers yield a high degree of neuronal differentiation and network formation in vitro, suggesting it as a promising platform for stimulating growth of neurological tissue. Simkova et al. (113) demonstrated a system in which NDs interact with the BBB and potentially trigger increased downstream release of interlukin-6 and interferon-gamma.

#### Wound healing

Given the antibiotic potential of conjugated NDs, enhanced wound healing represents a natural avenue of exploration. Additionally, ND conjugates can generally penetrate deeper into the dermal layers, improving the performance of wound healing agents (114).

For example study, Zeng et al. (115) developed a silver-polydopamine ND hydrogel that demonstrated sustained antibiotic qualities and enhanced wound healing in mice when compared with both control gels and PDA-mediated NDs without the silver augmentation.

In a particularly novel configuration of NDs, Khalid et al. (2020) demonstrate a three-fold system for wound healing that could be deployed in a single batch of NDs. The team prepared a hybrid ND-silk membrane. Conjugation with NDs not only increased the silk fibers’ thermal stability but also led to the retention of fluorescence and antibacterial qualities. The fluorescent NDs served as a nanothermometer to assess any temperature changes suggestive of infection or inflammation, while the silk fibers compensated for deficiencies in the wound area's extracellular matrix and accelerated the healing process. These NDs also retained biocidal propensity for Gram-negative bacteria, such as *Escherichia coli* and *Pseudomonas aeruginosa* (99). Abraham et al. (116) developed a similar ND-silk system to monitor and enhance wound healing but functionalized the NDs with curcumin, one of the active ingredients in turmeric known to have anti-inflammatory and antibiotic properties.

Additional approaches have conjugated NDs with polycaprolactone, a well-known candidate for tissue scaffold in wound healing, and demonstrated superior moisture control and thermal stability when compared with polycaprolactone alone (15, 94).

Notwithstanding these advances, additional approaches have still sought to leverage ND conjugates that enhance biocompatibility and performance of existing wound treatments, such as polypropylene (hernia) mesh (94, 117), and next-generation sutures with temperature sensing and antibiotic properties (118). In a particularly novel approach, Neuhoferova et al. (2017) developed a biocompatible wound dressing that delivers locally controlled release of siRNA targeting MMP-9 from a scaffold of degradable nanofibers. When tested in animal models of diabetes mellitus, the results showed favorable outcomes and accelerated healing with low-to-no toxicity on previously nonhealing wounds (119).

#### Bone regeneration

NDs have shown the capacity to enhance osteogenicity at specific sites. Rifai et al. (120) demonstrated that titanium-based implant material dipped in an aqueous suspension of unconjugated NDs increased bone and dermal tissue growth in vitro over controls. The team's work also showed that the ND coating simultaneously discouraged growth of *S. aureus*, which suggests an additional benefit of reduced infection at the site of implantation. Similar studies on SaOS-2 cell lines have yielded equally promising results for targeted/site-specific bone regeneration (121).

Another approach under investigation includes incorporation of phospholipid-conjugated NDs into poly-L-lactic acid (PLLA), which is itself an attractive media for tissue scaffolding because of its ability to be degraded in vivo and subsequently metabolized into carbon dioxide and water. Shuai et al. (122) reported that adding phospholipid-NDs into PLLA both strengthened the base scaffold and improved cellular adhesion, making the material an attractive candidate for bone tissue engineering.

Several more studies have reached similar conclusions about the capacity for NDs to enhance bone growth and stabilize scaffolding (123–126).

#### Dental regeneration

Lisik and Krokosz (2021) report that NDs also have been under investigation in dental implantology to both direct bone growth and reduce inflammation that can lead to periodontitis. A recent review of ND technology for maxillofacial pathologies and cancers cites current research in ND applications ranging from cavity (caries) prevention and restoration of decayed tooth enamel to enhanced osteointegration of dental implants to regeneration of lost periodontal tissue (127). NDs are even being investigated as a potential augmentation and enhancement for gutta percha, the filler material used to replace extracted dental pulp after root canal. Lee et al. (128) modified gutta percha with amoxicillin-functionalized NDs that were designed to reduce infection and enhance success rates.

## Conclusion

### Discussion

This review highlights some of the latest developments and investigations of NDs for the enhancement of our understanding of human biology and improvement in human health. Among emerging carbon nanomaterials—such as nanotubes, dots, and fullerenes—NDs have captured the imagination thanks to their unique properties, including superior biocompatibility, high chemical stability, and tunable surface chemistry (129). NDs have shown promise in far-reaching bioapplications that range from novel drug transporters across the BBB to stimulants for solid organ tissue regeneration to intracellular monitors of organelle activity and protein transport.

### Challenges and limitations

Despite their many advantages, certain challenges and limitations remain in the implementation of NDs in patient care. Namely, the effect of long-term exposure to NDs and their various functionalizations is an open question (23, 130). Of particular note, NDs to date have largely been studied either in vitro or in rodent models of disease and health, leaving the cytotoxicity of NDs in humans a critical point of investigation.

Additionally, consistent and reproducible synthesis of NDs is possible, but scalable systems to achieve this remains a challenging proposition (131). For example, large volumes of NDs can be produced in the detonation process, but the diameters of individual NDs can vary by several orders of magnitude within an individual batch. Conversely, CVD synthesis can yield NDs of a consistent size, but the overall diameter of the yield is generally larger than those produced through detonation. It is also noteworthy that both CVD and HPHT methods often involve grinding, leading to nonuniform sizes (132–134).

### Recommendations for future research

How NDs activate and affect the immune system remains an important unanswered question (34), as does their overall toxicity in humans. Additionally, it will be important to characterize the persistence of NDs and their impacts (if any) on sensitive ecosystems, such as aquatic life.

Synergistic interactions of NDs with other nanomaterials represents another exciting avenue for future study. So, too, does the prospect of resolving nanoscale interactions with quantum sensing. As our understanding of NDs matures along with other nanomaterials, it will be interesting to see how these novel materials can be deployed together as part of larger systems designed to prevent, diagnose, and treat disease. The future of ND research also holds exciting prospects in the realm of quantum sensing. NDs, particularly those containing nitrogen-vacancy (NV) centers, exhibit unique quantum properties that make them exceptional candidates for highly sensitive sensing applications. Leveraging these quantum features, ND-based sensors could usher in a new era of ultraprecise measurements, with potential applications spanning quantum computing, medical diagnostics, and environmental monitoring (135–137).
